# Hydrobromate of Caffein

**Published:** 1885-12-20

**Authors:** 


HYDROBROMATE OF CAFFEIN.—The enterprising house of W. R. Warner & Co. has recently added to its list of valuable preparations an effervescing salt of hydrobromate of caffein and bromide of potassium. It is admirable. We have tested a sample sent us, with most gratifying results, in a severe case of nervous headache. It is a pleasant and refreshing draught, acceptable to the taste, and gives prompt relief to the headache, and allays also the distressing nausea usually found in these cases.

THE SURGEON-GENERAL reports that during the past fiscal year 41,714 patients received relief through the Marine Hospital Service, and that efficient methods have been used to prevent the introduction of cholera into the United States.

